# Assessing the Use of Oxford Nanopore Technologies Sequencing for Detection of Stable *Wolbachia* Bacterial Strain in Symbiosis in Wild *Anopheles* Mosquitoes From the WHO African Region

**DOI:** 10.1111/1462-2920.70435

**Published:** 2026-09-30

**Authors:** Laura Chatterley, Nicola Hull, Isabel Hughes, Seynabou Sougoufara, Owain Meek, Murat Ceyran, Vishaal Dhokiya, Janvier Bandibabone, Cyprian Adala, Elin Cunningham, Shehu Shagari Awandu, Helene Kaminski‐Nenkam, Antonio Nkondjio, Grant L. Hughes, Eva Heinz, Thomas Walker

**Affiliations:** ^1^ School of Life Sciences The University of Warwick Coventry UK; ^2^ Strathclyde Institute of Pharmacy and Biomedical Sciences University of Strathclyde Glasgow Scotland; ^3^ Departments of Vector Biology and Tropical Disease Biology, Centre for Neglected Tropical Diseases Liverpool School of Tropical Medicine Liverpool UK; ^4^ Laboratoire D'entomologie médicale et Parasitologie Centre de Recherche en Sciences Naturelles (CRSN/LWIRO) Bukavu Democratic Republic of the Congo; ^5^ Department of Biological Sciences Jaramogi Oginga Odinga University of Science and Technology Bondo Kenya; ^6^ Laboratoire de Recherche sur le Paludisme Organisation de Coordination Pour la Lutte Contre Les Endémies en Afrique Centrale (OCEAC) Yaoundé Cameroon

**Keywords:** *Anopheles* mosquitoes, Flongle, malaria, MinION, nanopore sequencing, Oxford nanopore technologies (ONT), *Wolbachia*

## Abstract

Malaria remains a significant global health challenge with 263 million cases in 2023. *Anopheles* insecticide resistance threatens current vector control methods, and novel strategies are crucial to combat malaria transmission. *Wolbachia*, an endosymbiotic bacteria that can invade mosquito populations and inhibit pathogen replication, has only recently been shown as a high‐density symbiont in two wild *Anopheles* species. Detection of *Wolbachia* in symbiosis with *Anopheles* mosquitoes has relied on PCR amplification of *Wolbachia* genes and/or Sanger and Illumina sequencing. This study assesses Oxford Nanopore Technology (ONT) to detect *Wolbachia*, which could facilitate screening for symbiosis with wild *Anopheles* mosquitoes in endemic settings. We used 46 samples; 10 were pooled for a single MinION flow cell run and the rest processed individually using Flongle flow cells, comparing three mosquito species with three kit combinations. Using the ONT Field sequencing kit, there were 13X more *Wolbachia* reads in *An. demeilloni* (genuine symbiosis) than *An. gambiae* (no evidence of *Wolbachia*). The ONT 16S barcoding kit and Rapid barcoding kit were then assessed using more economical Flongle flow cells. *Wolbachia* was successfully identified in *An*. *demeilloni* and *An. moucheti* but not in *An. gambiae*. This study demonstrates the feasibility of ONT for field‐based detection of *Wolbachia*.

## Graphical abstract

This study assesses Oxford Nanopore Technology sequencing to detect *Wolbachia* in *Anopheles* mosquitoes. To do this, samples were analysed using the ONT field sequencing kit and sequenced using a minION flow cell and the ONT 16S barcoding kit and Rapid barcoding kit sequenced using Flongle flow cells. This aims at providing a streamlined methodology to facilitate screening for *Wolbachia* symbiosis in wild *Anopheles* mosquitoes in field‐based settings.

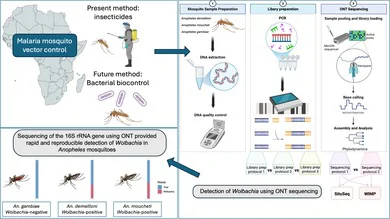

## 1 Introduction

Malaria is caused by *Plasmodium* parasites transmitted through the bites of infected *Anopheles (An.)* mosquitoes and there were an estimated 263 million malaria cases and 597,000 deaths globally in 2023 (WHO 2024). Despite encouraging progress in vaccine development, insecticide resistance has reduced the effectiveness of traditional vector control. Bacteria that naturally reside within *Anopheles* mosquitoes have been shown to inhibit *Plasmodium* but the major hurdle has been the lack of a natural mechanism to spread bacteria through mosquito populations (Dennison et al. 2016; Zhang and Dimopoulos 2026). *Wolbachia* endosymbiotic bacteria can invade mosquito populations through phenotypic adaptations such as cytoplasmic incompatibility (CI), which results from the sterility of progeny from matings between *Wolbachia*‐infected males and uninfected females (all other crosses are compatible), providing a natural drive mechanism. In *Aedes aegypti* mosquitoes that have been artificially transinfected, *Wolbachia* both invade wild populations and inhibit arbovirus transmission (Walker et al. 2011; Hoffmann et al. 2011; Utarini et al. 2021; Frentiu et al. 2014; Joubert et al. 2016). Integrated *Anopheles* vector management requires the development of novel control strategies that can work synergistically with existing methods. An attractive option would be *Wolbachia*‐based biocontrol for *Anopheles*. *Wolbachia*‐based biocontrol for *Aedes* is safe for humans and already widely accepted. Importantly, it also does not require complex, logistically challenging and highly infrastructure‐dependent distribution campaigns or specific assessments to target specific population groups. For replacement strategies only small release numbers are needed making this strategy sustainable, eco‐friendly and without bias against specific population groups or hard‐to‐reach settlements. Although the World Mosquito Program states that ‘*Wolbachia* can sustain itself in mosquito populations without continual reapplication, making this a long‐term, self‐sustaining approach’, further studies are needed to determine the long‐term cost effectiveness of this approach, that likely also differ between implementation locations. Furthermore, dengue suppression via *Wolbachia*‐infected *Ae. aegypti* release is the only method that has been shown to effectively reduce incidence across multiple countries, giving great hope that this could be effective against *Anopheles* mosquitoes for malaria control.

Translating either population replacement or suppression to *Anopheles* mosquitoes is not straightforward but mathematical models have already been developed to provide qualitative insights for designing optimal *Wolbachia* release strategies (Andreychuk and Yakob 2022; Florez et al. 2023). The *w*AlbB strain was transferred from *Ae. albopictus* into *An. stephensi* and resulted in significant *Plasmodium falciparum* inhibition and CI induction (Bian et al. 2013), but associated mosquito fitness costs prevented further progression to field release trials. Recently the *w*Pip strain from *Culex quinquefasciatus* mosquitoes has been stably introduced into *An. stephensi* under laboratory conditions and despite high‐density infections there was minimal impact on mosquito fitness (Liang et al. 2024) giving hope that this strain may provide more optimal phenotypic effects for release strategies. Given the technically challenging method of embryo microinjection to establish stable mosquito lines, adaptation of *Drosophila Wolbachia* strains to *Aedes* cell lines or using strains from closely related mosquito species were key reasons for successful *Ae. aegypti* transinfection (Iturbe‐Ormaetxe et al. 2011). Optimal candidate strains for species in the *Anopheles gambiae* complex or *Anopheles funestus* would be from within the *Anopheles* genera but historically *Wolbachia* was thought to not occur naturally within wild *Anopheles* populations. We have recently discovered strains in genuine endosymbiosis in *An. moucheti* (*w*AnM) and *An. demeilloni* (*w*AnD) which were visualised in mosquito ovaries (Walker et al. 2021). Our analyses also highlight that there is no evidence for *Wolbachia* in genuine symbiosis within major malaria vectors. Despite *Wolbachia* strains in the *An. gambiae* complex being at the threshold of detection, *Wolbachia* in *An. gambiae* and *An. coluzzii* have been shown to significantly reduce *Plasmodium* in naturally occurring populations (Gomes et al. 2017; Baldini et al. 2014).

For *Wolbachia* to be used as a successful control strategy, strains must be identified, characterised for phenotypic effects and monitored in natural *Anopheles* populations from malaria‐endemic countries. Currently surveillance detection methods used for *Wolbachia* include PCR amplification of *Wolbachia* specific genes or visualisation such as fluorescent in situ hybridisation. However, these are often expensive, time consuming and unless previous work has demonstrated evidence of *Wolbachia* strains in *Anopheles* populations, can result in insignificant evidence of a genuine endosymbiosis. Therefore, sequencing methods have been used to provide stronger evidence for the presence of genuine high‐density *Wolbachia* strains in *Anopheles* mosquito populations (Walker et al. 2021). Illumina or sanger sequencing have limited use due to high cost, the need for significant infrastructure or shipment of samples to sequencing facilities out of country, which can be logisitically challenging. Given these limitations, we explored the use of Oxford Nanopore Technologies (ONT) as a tool to identify natural *Wolbachia* strains in symbiosis in *Anopheles* populations given this sequencing method is rapid and can be undertaken with minimal equipment, opening the possibility of sequencing in malaria‐endemic field settings (Girgis et al. 2023). The MinION sequencer offers the possibility of being deployed in less‐developed laboratories across the WHO African region, empowering local facilities with advanced capabilities for research and the ability to perform ‘in house’ sequencing negating the need to send samples to other research institutes or commercial providers. Our study evaluated the feasibility of ONT for *Wolbachia* strain detection in *Anopheles* mosquitoes. Flongle flow cells have a maximum of 126 sequencing channels as opposed to the 512 maximum channels available on a standard MinION flow cell, therefore producing significantly less data output. Using more economical Flongle flow cells, we compared ONT sequencing using the portable Mk1b device across multiple *Anopheles* species comparing two library preparation kits: the Rapid PCR Barcoding Kit 24 V14 and 16S Barcoding Kit 24 V14. Our results showed consistent detection of *Wolbachia* within the two stably infected species (*An. demeilloni* and *An. moucheti)* but no evidence of *Wolbachia* strains in *An. gambiae*.

## 2 Methods

### 2.1 Sample Collection and Storage

Adult *Anopheles* mosquitoes of various species were collected from the DRC, Kenya and Cameroon with location and year of collection shown in Table 1. In Cameroon, adult female *Anopheles moucheti* were collected in 2019, 2022 and 2024 using human landing catches (Walker et al. 2021) from Olama Village (3.4125, 11.2841) and transported to OCEAC (Yaoundé) at 4°C for morphological identification. In Kenya, *Anopheles demeilloni* samples were collected in 2019 from Bigege Village (−0.5951053, 34.7488466) in Kisii, Western Kenya, using CDC light traps and transported to KEMRI (Kisumu) for morphological identification. In DRC, *Anopheles demeilloni* samples were collected during ongoing entomological surveys in 2019 and 2024 using manual aspiration from Lwiro (−2.244097, 28.815232) and Maziba (−2.241816, 28.800961*)* in the Sud Kivu region of eastern DRC. Morphologically identified adult females from all collection locations were placed individually in 1.5 mL Eppendorf tubes, transported to the University of Warwick in temperature‐controlled conditions and stored at −70°C prior to DNA extraction. Surface sterilisation using 70% ethanol and sterilised water washes was undertaken for all samples prior to DNA extraction.

**TABLE 1:** Species collection information and pool size of *Anopheles* mosquito genomic DNA used for library preparation using the ONT Field sequencing kit, ONT Rapid PCR Barcoding Kit 24 V14 and 16S Barcoding Kit 24 V14.

DNA extract sample	Mosquito species	Collection location	Collection year	Pool size
AN.DEM.DRC.2022.P2	*Anopheles demeilloni*	Maziba, DRC	2022	2
AN.DEM.DRC.2022.P4	*Anopheles demeilloni*	Maziba, DRC	2022	4
AN.DEM.DRC.2022.P10	*Anopheles demeilloni*	Maziba, DRC	2022	10
AN.GAM.DRC.2022.P2	*Anopheles gambiae*	Lwiro, DRC	2022	2
AN.GAM.DRC.2022.P4	*Anopheles gambiae*	Lwiro, DRC	2022	4
AN.GAM.DRC.2022.P10	*Anopheles gambiae*	Lwiro, DRC	2022	10
AN.DEM.KENYA.2019.P1	*Anopheles demeilloni*	Kisii, Kenya	2019	1
AN.DEM.DRC.2023.P3	*Anopheles demeilloni*	Lwiro, DRC	2023	3
AN.DEM.DRC.2024.P1	*Anopheles demeilloni*	Lwiro, DRC	2024	1
AN.MOU.CAM.2024.P1	*Anopheles moucheti*	Olama Village, Cameroon	2024	1
AN.MOU.CAM.2019.P1	*Anopheles moucheti*	Olama Village, Cameroon	2019	1
AN.MOU.CAM.2019.P1.2	*Anopheles moucheti*	Olama Village, Cameroon	2019	1
AN.GAM.DRC.2023.P3	*Anopheles gambiae*	Lwiro, DRC	2023	3
AN.GAM.DRC.2023.P3.2	*Anopheles gambiae*	Lwiro, DRC	2023	3

*Note:* Where possible the same DNA samples were used for both the ONT Rapid PCR Barcoding Kit 24 V14 and 16S Barcoding Kit 24 V14 kit to allow direct comparison although due to limited volumes of DNA this was only possible for AN.DEM.KENYA.2019.P1, AN.DEM.DRC.2023.P3, AN.DEM.DRC.2024.P1 and AN.MOU.CAM.2019.P1.

Abbreviations: AN, *Anopheles*; DEM, *demeilloni*; GAM, *gambiae*; MOU, *moucheti*; *P*, pool size.

### 2.2 DNA Extraction, Quantification and *Wolbachia* Screening

Genomic DNA (gDNA) was extracted from adult female *Anopheles* mosquitoes using Qiagen DNeasy Blood and tissue kits according to manufacturer's protocols. Homogenisation of whole mosquitoes prior to DNA extraction was achieved using Qiagen Tissue lyser II (Qiagen, The Netherlands) at a speed of 25 Hz for 5 min. Samples were used for library preparation as either individuals or in pools of three mosquitoes post‐extraction if insufficient DNA concentration was obtained for individual samples (Table 1). Before beginning ONT library preparation and standardising the concentration of DNA, DNA extracts were quantified using a Qubit 4 fluorometer (Invitrogen, Thermo‐fisher scientific, Massachusetts, USA). For this 2 μL of DNA extracts were individually combined with 198 μL of the 1X Invitrogen dsDNA high sensitivity assay (cat no. Q33231) and placed in the Qubit 4 fluorometer to determine the concentration of DNA. This process was used for all DNA quantification stages throughout the study including post PCR quantification. All mosquito extracts were subject to *Wolbachia* PCR analysis as previously described (Walker et al. 2021) to confirm the presence of *Wolbachia* in *An. moucheti* and *An. demeilloni* extracts and no *Wolbachia* detection in *An. gambiae ss* (negative control sample).

### 2.3 ONT Field Sequencing Kit (SKU: SQK‐LRK001) Library Preparation

DNA extracted from pools of mosquitoes (2, 4, 10 and 12 mosquitoes/pool) was analysed for total DNA to determine suitability for the Field sequencing kit (Table 1). Mosquito pool sizes of > 10 only resulted in sufficient genomic DNA (gDNA) to proceed with library preparation given the sequencing kit requirements of 400 ng of gDNA in 10 μL. In brief, 10 μL of gDNA was added to the Fragmentation Mix and then mixed, incubated at room temperature for 1 min and then at 80°C for 1 min. Ten microlitres of fragmented DNA was then added to the rapid adapter and mixed before being incubated at room temperature for 5 min then immediately loaded onto the ONT flow cell.

### 2.4 ONT Rapid PCR Barcoding Kit 24 V14 (SQK‐RPB114.24) Library Preparation

Individual mosquito gDNA was prepared by diluting 1–5 ng of DNA in nuclease free water to a volume of 3 μL followed by combining with the fragmentation mix (FRM) and placed in a thermocycler (T100 Thermocycler, BioRad, California, USA) at 30°C for 2 min and then 80°C for 2 min. PCR was undertaken by combining the FRM/gDNA with NEB LongAmp Taq 2X master mix (cat no. M0287S) and each sample was combined with a different barcode (RLB) and underwent PCR amplification in a Bio‐Rad T100 Thermocycler (California, USA) with initial denaturation (95°C for 3 min), followed by 25 cycles of 95°C for 15 s, 55°C for 15 s and 65°C for 6 min with a final extension step of 65°C for 6 min. Following lack of sufficient band visualised on 1% agarose gel (Invtrogen, Thermo Fisher Scientific, USA) after several preliminary trial PCRs at the recommended 14 cycles, we increased to 25 cycles to continue to library preparation. After PCR, EDTA was added to the reaction and DNA quantification was undertaken using an Invitrogen Qubit 1X dsDNA High Sensitivity (HS) assay (cat no. Q33232) and an Invitrogen Qubit 4 fluorometer. Each barcoded sample was then pooled in an equimolar ratio and combined with AMPure XP beads (AXP) at a 0.6× volume ratio. Samples were incubated on a hula mixer (TR‐200 Rotator, Cole Parmer, Illinois, USA) and then pelleted on a magnetic rack (Ambion Magnetic Stand‐96 AM10027, Invitrogen, Thermo‐fisher scientific, Massachusetts, USA) and supernatant removed and discarded. Pellets were then washed twice with 80% ethanol solution and resuspended in elution buffer (EB) and then placed back on the magnetic rack where the eluate was retained. After the second quantification with the Invitrogen Qubit 4 fluorometer, 5–25 fmol of sample was combined with EB to make up 5.5 μL and combined with 0.5 μL of diluted rapid adapter (dRA). DNA libraries were then combined with sequencing buffer (SB) and library beads (LB).

### 2.5 16S Barcoding Kit 24 V14 (SQK‐16S114.24) Library Preparation

Extracted gDNA from individual mosquitoes was prepared by diluting 10 ng of DNA in 15 μL of nuclease free water and then the diluted DNA was combined with NEB LongAmp Taq 2X master mix and respective 16S barcodes. Samples were submitted to PCR amplification in a Bio‐Rad T100 Thermocycler (California, USA) with initial denaturation (95°C for 1 min), followed by 25 cycles of 95°C for 20 s, 55°C for 30 s and 65°C for 2 min with a final extension step of 65°C for 5 min. A volume of 4 μL of EDTA was added to each barcoded sample before pooling and mixing with AMPure XP beads in a 0.6× ratio. Samples were incubated on a hula mixer (TR‐200 Rotator, Cole Parmer, Illinois, USA) and then pelleted on a magnetic rack (Ambion Magnetic Stand‐96 AM10027, Invitrogen, Thermo‐fisher scientific, Massachusetts, USA) and supernatant removed and discarded. The pellets were then washed twice with 80% ethanol solution and resuspended in elution buffer (EB) and then placed back on the magnetic rack where the eluate was retained. After the second quantification with the Qubit 4 fluorometer, 5–25 fmol of sample was combined with EB to make up 5.5 μL and combined 0.5 μL of diluted rapid adapter (dRA). DNA libraries were then combined with sequencing buffer (SB) and library beads (LB).

### 2.6 ONT Sequencing

The Oxford Nanopore Technologies MinION Mk1C (a self‐contained device that combines the hardware for running nanopore sequencing experiments with fully integrated compute used for base calling and onward analysis) was first tested using pooled DNA libraries produced using the field sequencing kit (SKU: SQK‐LRK001) on MinION flow cells (FLO‐MIN004RA). A single flow cell run with a library prepared using the Rapid PCR Barcoding Kit 24 V14 (SQK‐RPB114.24) was tested on the Mk1C but failed. All further ONT sequencing was performed using Flongle flow cells (FLO‐FLG114) with a MinION Mk1B and flongle adapter powered by a Dell standard AIO desktop (210‐BFWY) computer with an operating system > 16 GB ram, a USB3 port and an internal or external storage of 1 TB. Individual singular samples from gDNA extracted from an individual *Anopheles* mosquito were first tested and gDNA sample numbers increased on a single Flongle flow cell once consistent sequencing outputs were obtained. This was executed by using the barcoding features of each kit, barcoding different DNA samples before entering a final pool for sequencing. This additionally provided quality control testing of accuracy when barcoding multiple replicates of the same gDNA sample.

### 2.7 Sequencing Analysis

Analysis of the raw sequencing data output was achieved using the EPI2ME agent and portal software application. Within the EPI2ME agent, the MinKNOW software automatically saved all output files including FASTQ files that contain the sequence of nucleotide bases to be analysed. After sequencing completion, an output of FASTQ files was sorted by MinKNOW into FASTQ pass and FASTQ fail and passed files inputted into the EPI2ME agent analysis platform and assessed using a What's In My Pot (WIMP) analysis for each individual run and split by barcode. Analysis was undertaken using the Centrifuge version 1.0.3‐beta classification method, then results were filtered and combined to determine the counts of reads at species‐level taxonomic assignments. For reads without a reliable species‐level assignment, these were placed at ranks higher in the taxonomic trees but if reads were deemed unreliable, they were not included and reported as unclassified. A ‘Fastq 16S’ Bacterial analysis was employed for all runs using the 16S Barcoding Kit 24 V14 (SQK‐16S114.24) but all failed with no reports available. Finally, phylogenetic analysis and tree construction was undertaken from WIMP analysis within the EPI2ME platform. Generated FASTQ files were also analysed using a SituSeq pipeline (Zorz et al. 2023) employing *DADA2 (*Callahan et al. 2016 *)*. Following this pipeline, primers were trimmed from all reads, reads were then filtered and trimmed based on observed quality before being taxonomically assigned using Silva (Version 138.1 SSU) (Quast et al. 2013) and Relative abundance explored at the genus level via bar charts created in *ggplot2 (*Wickham 2016 *)*.

## 3 Results

### 3.1 Total Sequencing Data Output Comparison of Library Preparation Kits

Analysis of pooled mosquitoes (*n* = 10) revealed total reads for *An. demeilloni* (sample AN.DEM.DRC.2022.P10) was 139,950 compared to 72,659 for *An. gambiae* (sample AN.GAM.DRC.2022.P10) (Figure 1) when using the ONT Field sequencing kit libraries sequenced on the MinION Mk1C. The percentage of reads that were classified was similar (46.1%, 42.7%, respectively) and of those that were classified both samples showed bacterial reads at greater than 60%. The requirement to use pooled mosquitoes (> 10) to obtain sufficient gDNA and the discontinuation of both the field sequencing kit for library preparation and the MinION Mk1C devise prevented a more comprehensive analysis. Instead, we assessed ONT sequencing using two library preparation kits (Rapid PCR Barcoding Kit 24 V14 and 16S Barcoding Kit 24 V14) using the more economical flongle flow cells (FLO‐FLG114) and a Flongle adapter on a MinION Mk1B device on gDNA extracted from three *Anopheles* mosquito species: *An. gambiae*, *An. demeilloni* and *An. moucheti*. A total of 10/11 (91%) sequencing runs were successful with only a single run failing to produce any reads above the quality score threshold of nine (Table 2). The Rapid PCR Barcoding Kit library preparation allows sequencing of the whole genome in contrast to the 16S Barcoding Kit library preparation kit which only targets the ubiquitous bacterial *16S rRNA* gene. Overall, the Rapid PCR Barcoding Kit obtained a higher mean volume of data with 4/5 (80%) runs achieving over the theoretical max output of 2.6 GB for a Flongle flow cell (this was only achieved on one sequencing run for the 16S Barcoding Kit) (Figure 2A). On average the Rapid PCR Barcoding Kit generated 5.9 Gb of data compared to only 1.5 Gb of data for the 16S Barcoding Kit. A similar trend was observed for the total amount of reads produced with 148,034 for the Rapid PCR Barcoding Kit and 63,854 reads for the 16S Barcoding Kit (Figure 2B). However, the most successful run (no. 7) in terms of read number and data did result from the 16S Barcoding Kit 24 V14 producing 254,340 reads. Taxonomic classification of the resulting reads varied depending on which sequencing kit was used (Figure 3). However, *Wolbachia* was consistently in the most abundant for this sample (AN.MOU.CAM.2019.P1.2) regardless of sequencing kit.

**FIGURE 1:**
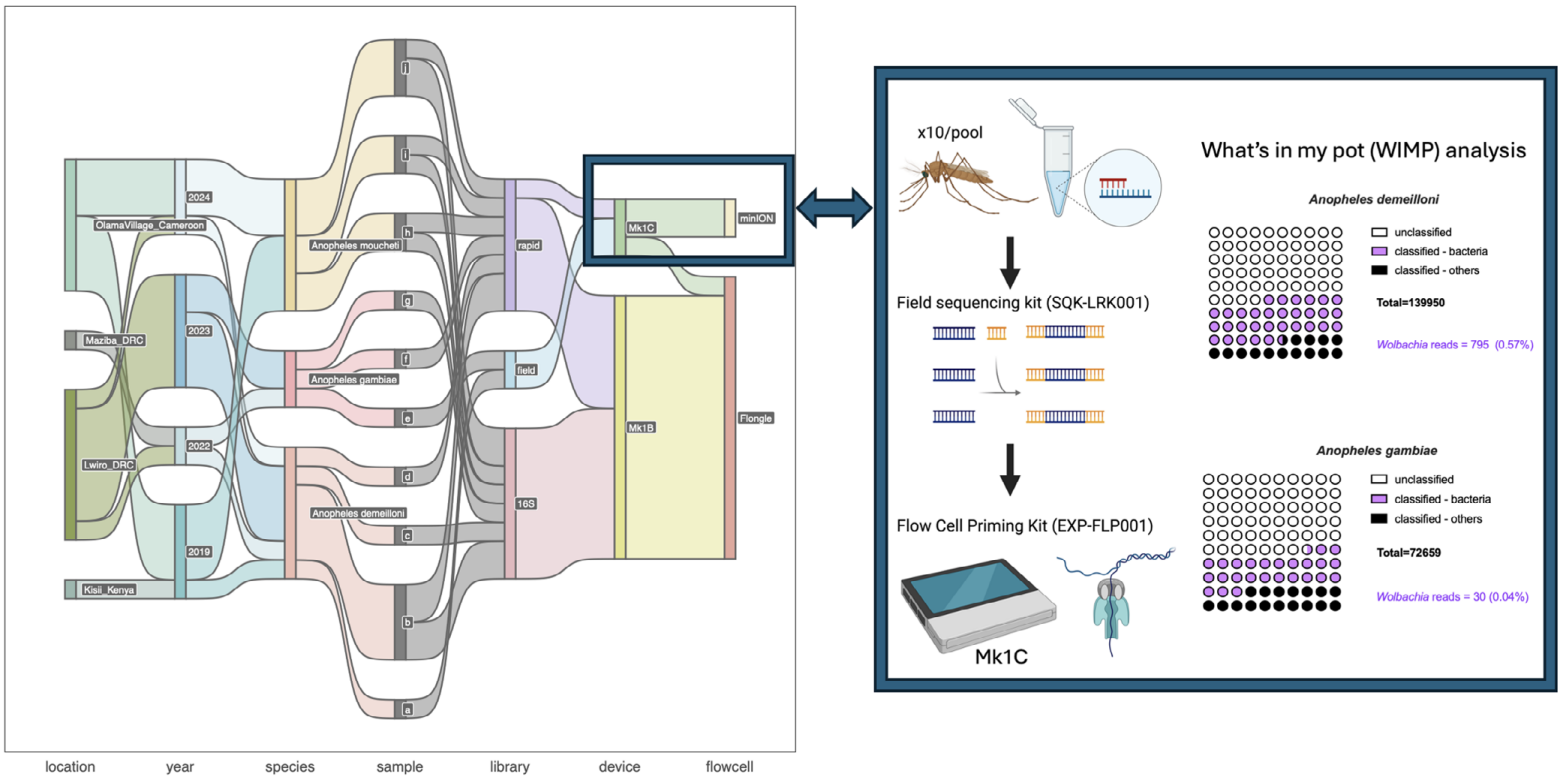
Sankey diagram to visualise the different samples and processes undertaken using ONT sequencing and results of ONT WIMP analysis of using the field sequencing kit library preparations (*n* = 10 mosquitoes/pool) from MinION (R9.4.1) sequencing on the Mk1C device. *Anopheles demeilloni* sample = AN.DEM.DRC.2022.P10, *Anopheles gambiae* sample = AN.GAM.DRC.2022.P10. Sankey diagram generated using ggsankey package in R.

**TABLE 2:** ONT sequencing runs using flongle flow cells (FLO‐FLG114).

Run	Library prep kit	Device	Species	Samples tested	Barcode	Total reads	Q score	*Wolbachia* reads
1	Rapid PCR	Mk1C	*An. demeilloni*	AN.DEM.DRC.2023.P3	RLB1	400,000	6.16	39
2	Rapid PCR	Mk1B	*An. moucheti*	AN.MOU.CAM.2024.P1	RLB2	6000	13.47	110
				AN.MOU.CAM.2024.P1	RLB4	5000	13.42	110
3	Rapid PCR	Mk1B	*An. demeilloni*	AN.DEM.KENYA.2019.P1	RLB5	5669	12.71	0
				AN.DEM.KENYA.2019.P1	RLB7	6701	12.54	2
4	Rapid PCR	Mk1B	*An. moucheti*	AN.MOU.CAM.2019.P1	RLB12	5000	13.1	29
				AN.MOU.CAM.2019.P1.2	RLB18	13,000	12.81	60
5	Rapid PCR	Mk1B	*An. demeilloni*	AN.DEM.DRC.2023.P3	RLB5	2905	13.25	1
				AN.DEM.DRC.2023.P3	RLB6	10,441	13.25	26
				AN.DEM.DRC.2023.P3	RLB7	3985	13.25	7
6	Rapid PCR	Mk1B	*An. gambiae*	AN.GAM.DRC.2023.P3	RLB12	12,393	12.03	0
7	16S	Mk1B	*An. moucheti*	AN.MOU.CAM.2024.P1	RLB1	7788	11.49	1189
				AN.MOU.CAM.2024.P1	RLB2	5901	11.49	757
				AN.MOU.CAM.2024.P1	RLB3	7972	11.49	1278
				AN.MOU.CAM.2024.P1	RLB4	10,008	11.49	2809
8	16S	Mk1B	*An. moucheti*	AN.MOU.CAM.2019.P1	RLB13	9709	12.4	5289
				AN.MOU.CAM.2019.P1	RLB15	17,689	12.4	14,111
				AN.MOU.CAM.2019.P1.2	RLB9	1649	12.4	1018
				AN.MOU.CAM.2019.P1.2	RLB 12	1831	12.4	1070
9	16S	Mk1B	*An. demeilloni*	AN.DEM.DRC.2023.P3	All RLBs	67	< 9	30
10*	16S	Mk1B	*An. demeilloni*	AN.DEM.DRC.2024.P1	All RLBs	0	0	0
11	16S	Mk1B	*An. demeilloni*	AN.DEM.DRC.2024.P1	RLB1, RLB2	2183	10.25	1246
12	16S	Mk1B	*An. gambiae*	AN.GAM.DRC.2023.P3.2	All RLBs	4491	12.62	0

*Note:* Samples tested were individually barcoded unless indicated and in some flow cell runs (e.g., Run 2) the same DNA sample was used in two individual barcodes and run in replicate on the same flow cell. * Asterisk indicates run failure.

**FIGURE 2:**
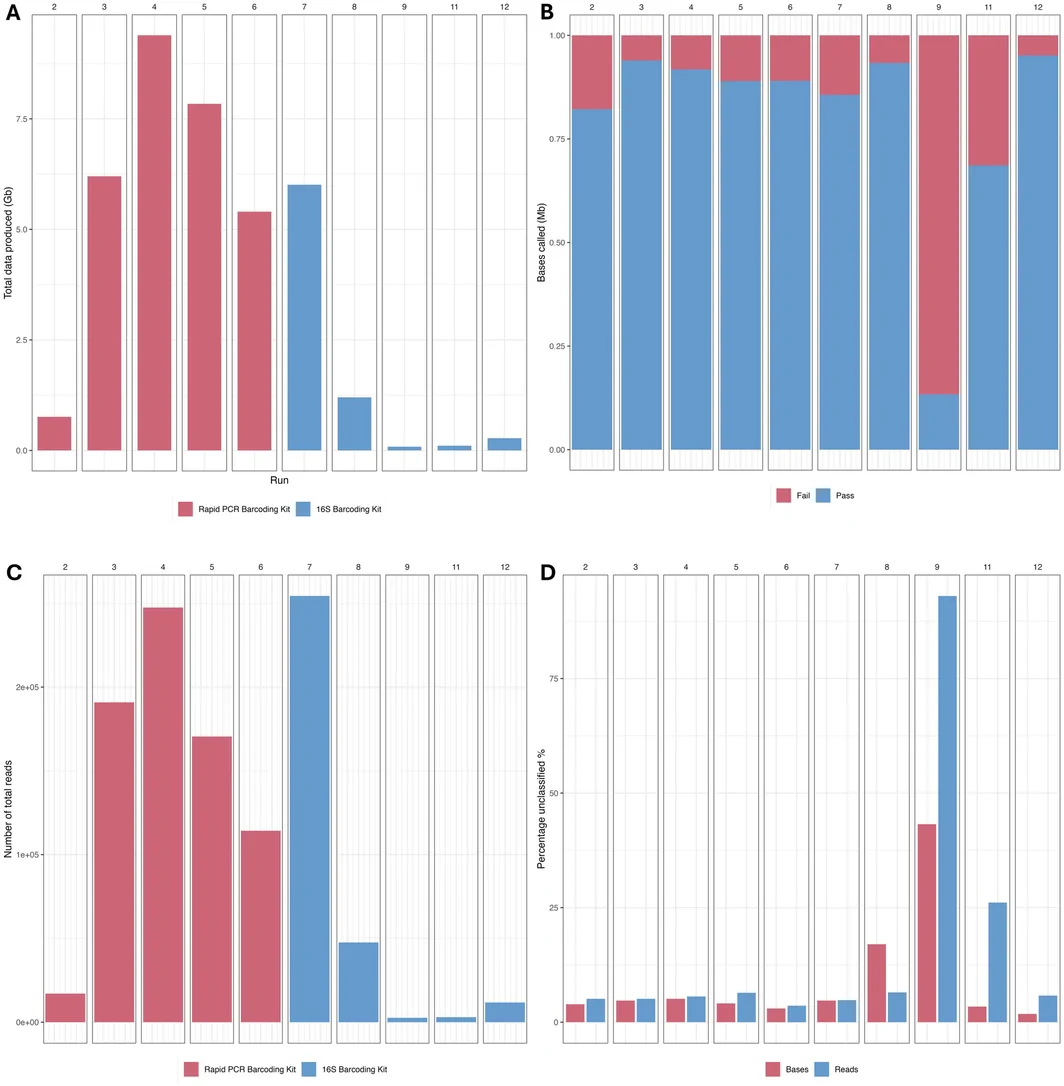
Evaluation of overall output ONT sequencing data for flongle flow cell runs on *Anopheles* gDNA samples. All bar graphs depict sequencing runs using two different barcoding kits: The Rapid PCR Barcoding Kit 24 V14 (runs 2–6) and the 16S Barcoding Kit 24 V14 (runs 7, 8, 9, 11, 12). Each run is represented by a vertical column. (A) Total data generated (GB). (B) Percentage of passed vs. failed bases called for each run. (C) Total number of reads obtained in each sequencing run. (D) Percentage of unclassified passed reads and unclassified passed bases for each run.

**FIGURE 3:**
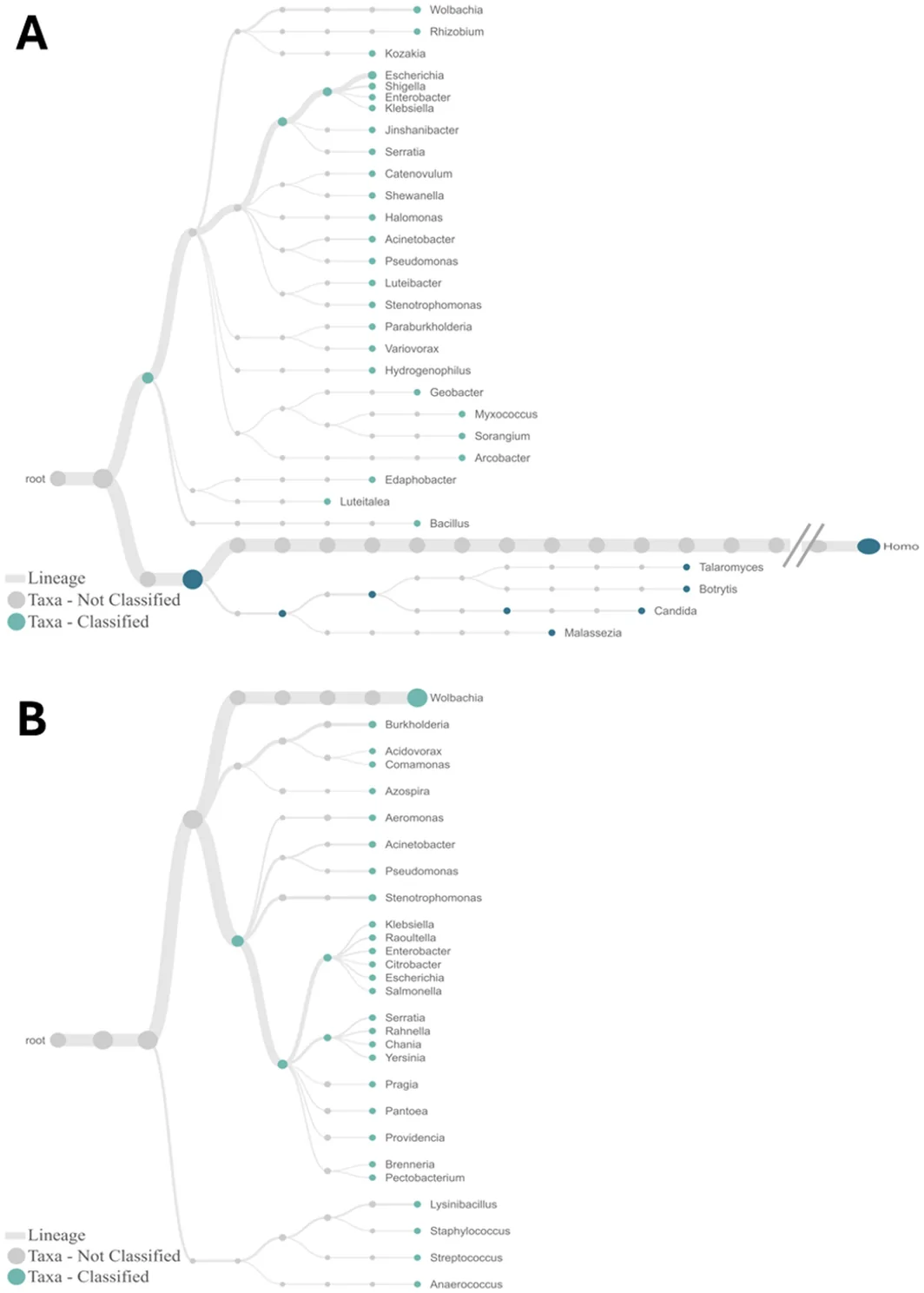
Taxonomic Classification of Microbial Communities from WIMP analysis of *An. moucheti* (sample AN.MOU.CAM.2019.P1.2). (A) Library prepared with the Rapid PCR Barcoding Kit 24 V14 (B) Library prepared with the 16S Barcoding Kit 24 V14. Both phylogenetic trees show the top 30 genera at the cut off abundance 0.1%.

### 3.2 Base Calling Failure Rates

The percentage of bases that were called successfully where a pass was considered as a minimum quality score of 9 was also assessed. On average 10.8% of base calling failed when using the Rapid PCR Barcoding Kit compared to 28.8% of base calling failed when using the 16S Barcoding Kit (Figure 2C). Flonge flow cell run nine had the poorest quality with 86% of bases called falling below the quality cut‐off score of nine. Removing run nine (which may have resulted from a technical error), the percentage base call failure rate for the 16S Barcoding Kit library preparations was still higher at 14.3%. Both Rapid PCR Barcoding Kit and the 16S Barcoding Kit library preparation methods utilised barcoding of mosquito gDNA samples, in cases where only one sample was used per run multiple barcodes of the same sample were used as per the protocol. On average the Rapid PCR Barcoding Kit had 5.16% and 4.16% unclassified reads and bases respectively (Figure 2D). In comparison, the 16S Barcoding Kit produced higher proportions of unclassified reads and bases at 27.24% reads and 14.02% bases unclassified. This demonstrates that a higher percentage of the sequencing data is lost due to failures in barcoding when using the 16S Barcoding Kit. The maximum samples running simultaneously was estimated to be four using flongle flow cells as beyond this we were not able to produce high enough read counts whereby results would be accepted using the quality cut off scores.

### 3.3 Comparison of ONT Sequencing

Sequencing data output from libraries generated from the field sequencing library kit (MinION flow cell—R9.4.1) and libraries generated from the Rapid PCR Barcoding Kit 24 V14 (Flongle flow cell ‐FLO‐FLG114) with identical sequencing run lengths of 24 h were compared. The MinION flow cell (R9.4.1) theoretically produces approximately 18.5 times the volume of data than the Flongle flow cell (FLO‐FLG114). However, when compared to the results achieved from the Flongle flow cell runs (Table 3), runs 4, 5 and 6 achieved greater volumes of data. However, this was consistent for all runs as sequencing data output showed significant variability from 0.1 to 9.39 Gb on average. In 4/5 (80%) of sequencing runs undertaken on libraries generated from the Rapid PCR Barcoding Kit and sequenced on the Flongle flow cell, more bases were produced than sequencing on the MinION flow cell. In contrast, libraries generated from the 16S Barcoding Kit only produced a greater number of bases than the MinION flow cell in one instance (Run 7). Although sequencing on the MinION flow cell achieved 100% of reads called this was not achieved in any of the Flongle flow cell runs, with some as low as 8.74% (Run 4) with the highest % reads called for a 24‐h run at 95.71%. The MinION flow cell also achieved a higher number of bases passed than any Flongle flow cell run and had a higher proportion of reads called and greater amount of passed called bases. However, sequencing on Flongle flow cells produce similar numbers of estimated bases, reads generated and total data produced.

**TABLE 3:** ONT sequencing output comparison for run duration of 24 h comparing three library preparation kits.

Sample(s)	Library prep kit	Estimated bases (Mb)	Reads generated	Total data (Gb)	Reads called (%)	Bases passed (Mb)	Bases failed (Mb)
AN.DEM.DRC.2022.P10 (*An. demeilloni*)	Field	290	251,010	5.3	100	86.08	171.72
AN.MOU.CAM.2024.P1 AN.MOU.CAM.2019.P1 (*An. moucheti*)	Rapid PCR	699	247,480	9.4	8.74	59.32	5.31
AN.DEM.DRC.2023.P3 x2 (*An. demeilloni*)	Rapid PCR	613	170,480	7.8	10.14	58.50	7.24
AN.MOU.CAM.2024.P1 x4 (*An. moucheti*)	16S	374	254,340	6.0	15.00	51.17	8.57
AN.DEM.DRC.2023.P3 x2 (*An. demeilloni*)	16S	6	2630	0.1	95.71	0.17	1.11

* MinION (R9.4.1) flow cell used in conjunction with field library preparation kit; remaining samples tested with Flongle (FLO‐FLG14) flow cell.

### 3.4 Overall Microbiome Relative Abundance

Analysis using WIMP workflow from the EPI2ME platform was utilised for libraries sequenced using the 16S Barcoding Kit to determine the relative abundance of bacteria in *An. moucheti*, *An. demeilloni* and *An. gambiae*. Initially the consistency of the microbiome profile using four replicate samples of *An. moucheti* (sample AN.MOU.CAM.2024.P1) was assessed with consistent profiles generated for each barcode (Figure 4A). Across all replicate libraries, the bacterial community composition appeared consistent, with *Wolbachia* being the most dominant bacteria. *Enterobacter*, *Escherichia* and *Klebsiella* also were significant bacteria genera in this sample. Following a SituSeq pipeline, taxonomy was assigned to *16S rRNA* reads using Silva database. The number of reads pre‐ and post‐ quality filtering can be found in Table 4 with all barcodes resulting in fewer than 10 initial reads removed. The same consistency as described previously was observed between different barcodes for sample AN.MOU.CAM.2024.P1 (Figure 4B).

**FIGURE 4:**
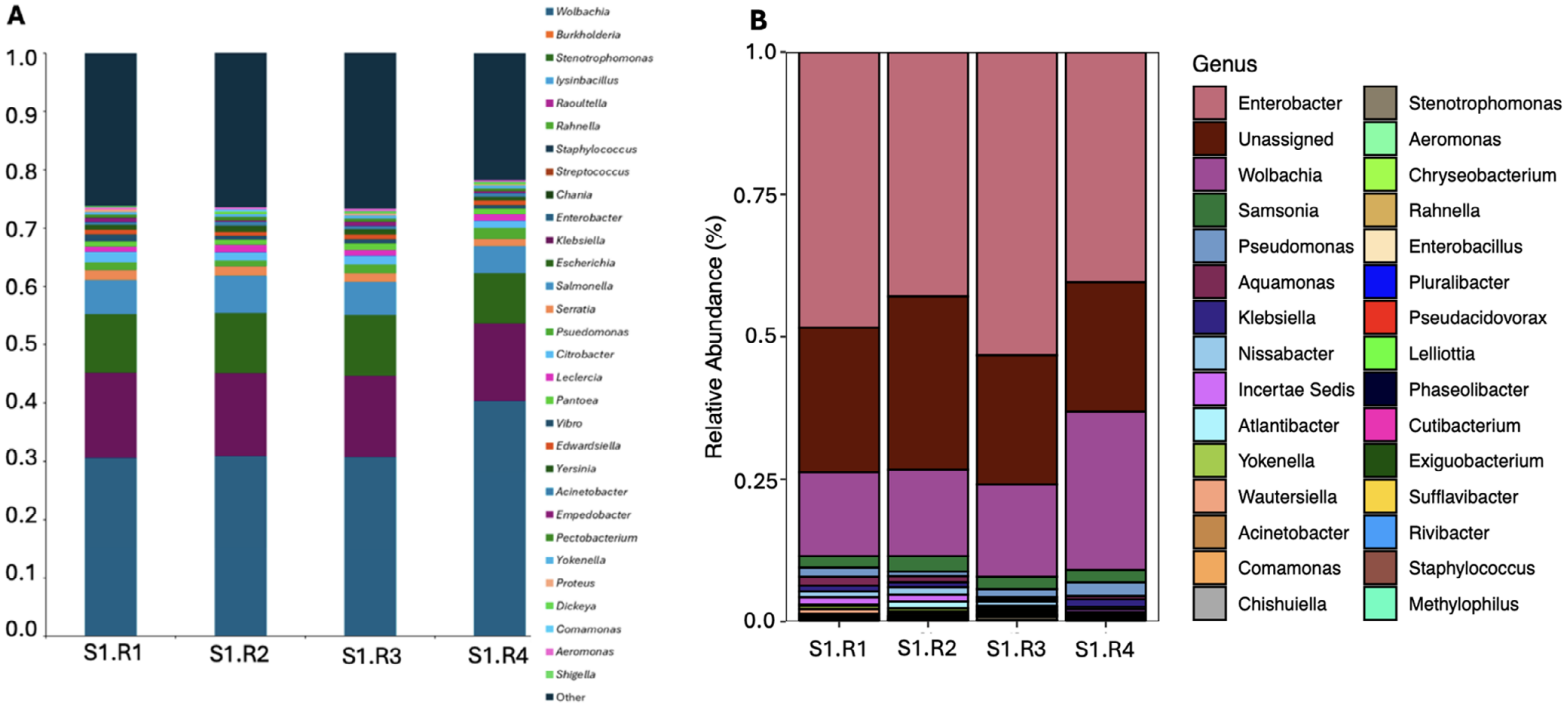
The microbial composition of *An. moucheti* (sample AN.MOU.CAM.2024.P1) across four sequencing replicates using the 16S rRNA Barcoding Kit 24 V14. Output was analysed using two different pipelines: (A) Epi2Me and (B) SituSeq. Bacterial genera are listed in order of relative abundance. ‘Other’ represents the sum relative abundance of bacterial genera that fell outside of the top 30 most abundant. ‘Unassigned’ refers to all reads that were not assigned to the genus level following the SituSeq pipeline.

**TABLE 4:** Number of reads generated per barcode using *16S rRNA* nanopore sequencing kit for individual *Anopheles* mosquitoes.

Species	Sample	Barcode	Input reads	Filtered reads
*An. moucheti*	AN.MOU.CAM.2024.P1	RLB1	1788	1433
		RLB2	2091	1653
		RLB3	3000	2459
		RLB4	3008	2425
		RLB9	1	1
		RLB16	1	0
	AN.MOU.CAM.2019.P1	RLB13	9709	9366
		RLB15	17,689	17,079
	AN.MOU.CAM.2019.P1.2	RLB09	1649	1576
		RLB12	1831	1739
*An. demeilloni*	AN.DEM.DRC.2023.P3	RLB17	104	84
		RLB18	88	72
		RLB19	36	36
		RLB20	40	28
	AN.DEM.DRC.2024.P1	RLB01	931	911
		RLB02	682	670
		RLB04	2	2
		RLB05	1	1
*An. gambiae*	AN.GAM.DRC.2023.P3.2	RLB17	3735	2322
		RLB18	448	252
		RLB24	47	21

*Note:* Input reads show the number of reads generated by ONT sequencing and filtered reads show those that passed SituSeq pipeline quality filters.

Taxonomic assignments were then assessed in terms of relative abundance (Figure 5) following the Epi2ME (Figure 5A) or SituSeq (Figure 5B) pipelines. There were several consistent findings between pipelines. *Wolbachia* dominated the *An. moucheti* and *An. demeilloni* microbiome and was not identified in the *An. gambiae* microbiome. Conversely, the genus *Asaia* had the highest relative abundance in *An. gambiae*, but was not identified in either of the other *Anopheles* species. There was a high relative abundance of *Enterobacter* in sample AN.MOU.CAM.2024.P1 observed in the output of both pipelines, with a slightly higher relative abundance estimated using the SituSeq pipeline. The single *An. gambiae* sample included (AN.GAM.DRC.2023.P3.2) was dominated by *Asaia* in both resulting analyses, however when assessing the SituSeq pipeline a high relative abundance of *Swaminathania* was also seen, which was not identified using Epi2ME analysis. When assessing the reads using the SituSeq pipeline, there was a relatively high proportion of unassigned reads in the majority of samples, with this group having the fourth highest overall relative abundance, however, this did vary between individual samples.

**FIGURE 5:**
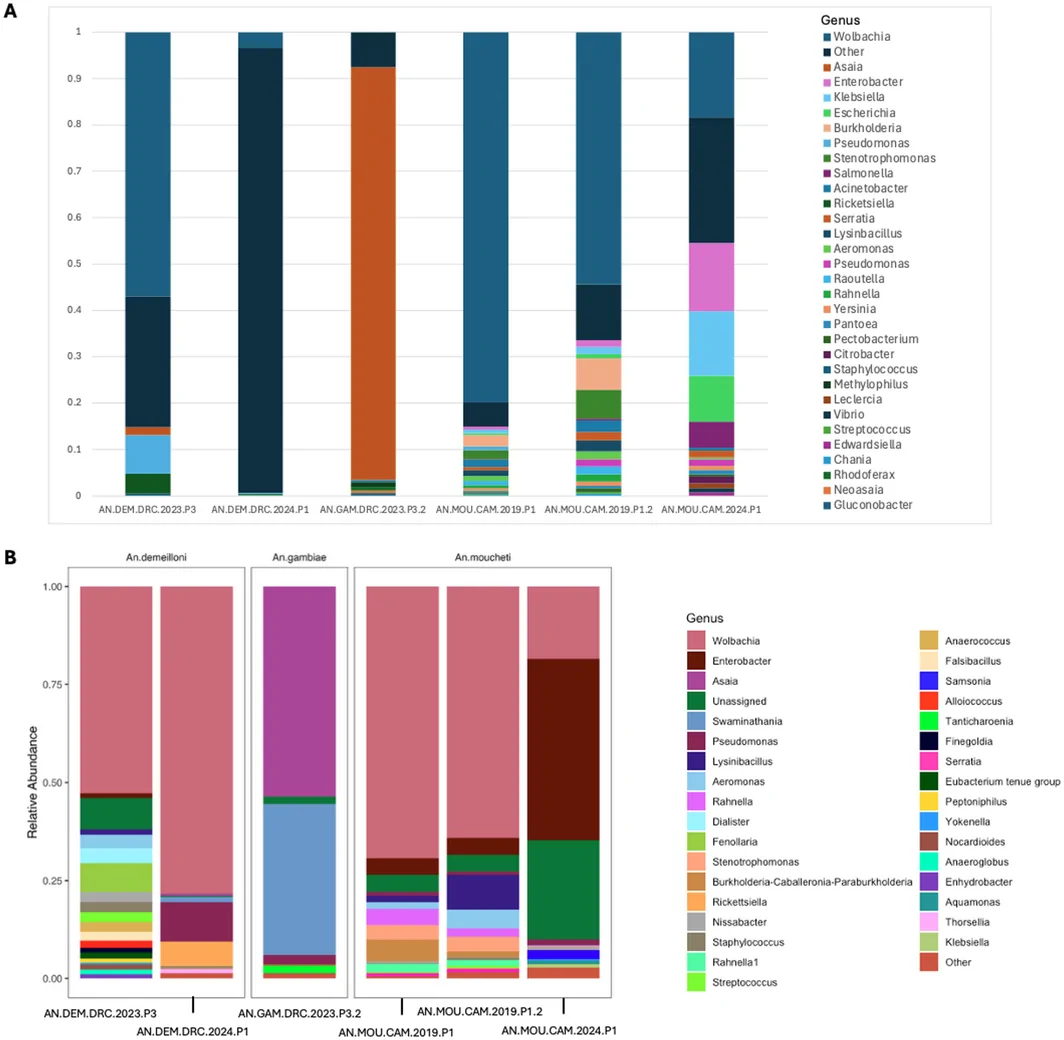
Relative abundance of bacterial genera within the *An. demeilloni*, *An. gambiae* and *An. moucheti* microbiome assessed using the 16S rRNA Barcoding Kit 24 V14. Output was analysed using two different pipelines: (A) Epi2Me and (B) SituSeq. Bacterial genera are listed in order of relative abundance. For plot A, ‘Other’ represents the sum relative abundance of bacterial genera that fell outside of the top 30 most abundant. In plot B, ‘Unassigned’ refers to all reads that were not assigned to the genus level following the SituSeq pipeline and ‘Other’ represents all bacterial genera that had a relative abundance of less than 1% of overall reads.

### 3.5 *Wolbachia* Detection Using ONT

Using a library generated from the Field sequencing kit sequenced on the MinION Mk1C, pooled *An. demeilloni* mosquitoes (sample AN.DEM.DRC.2022.P10) had total reads of 139,950 of which 69% were bacteria (Figure 1). The cumulative read count indicated the most abundant bacterial genera was *Escherichia* followed by *Wolbachia* (794 reads) which equated to 0.57% of total reads. Interestingly, the genus *Asaia* (known to compete with *Wolbachia* in *Anopheles* lab populations for colonisation of the germline (Hughes et al. 2014)), was also present with 182 reads. As this library preparation kit required pooling 10 mosquitoes to obtain sufficient gDNA, limited conclusions can be drawn in terms of co‐infection of *Asaia* and *Wolbachia* in individual *An. demeilloni* mosquitoes. Pooled *An. gambiae* mosquitoes (sample AN.GAM.DRC.2022.P10) resulted in total reads of 72,659 with 60% identified as bacteria. In contrast to *An. demeilloni*, only 30 *Wolbachia* reads were identified in *An. gambiae* which equated to only 0.04% of total reads. *Wolbachia*‐designated reads were ~13‐fold more abundant in *An. demeilloni than An. gambiae* which is consistent with other methods comparing *Wolbachia* densities in these two species such as quantitiave PCR and *16S rRNA* sequencing (Walker et al. 2021). When using flongle flow cells and libraries prepared using the Rapid PCR Barcoding Kit, WIMP analysis (Figure 6) resulted in an average of 42 *Wolbachia* reads were detected for *An. demeilloni* and *An. moucheti* (Table 2). In contrast, libraries prepared using the 16S Barcoding Kit averaged significantly more *Wolbachia* reads at 2038. In terms of abundance (*Wolbachia*/total reads), the 16S Barcoding Kit average was 0.42 compared to only 0.006 for the Rapid PCR Barcoding Kit library preparation method. Neither library preparation method used in combination with flongle flow cells resulted in any *Wolbachia* reads in *An. gambiae* confirming the infection status using conventional PCR based methods.

**FIGURE 6:**
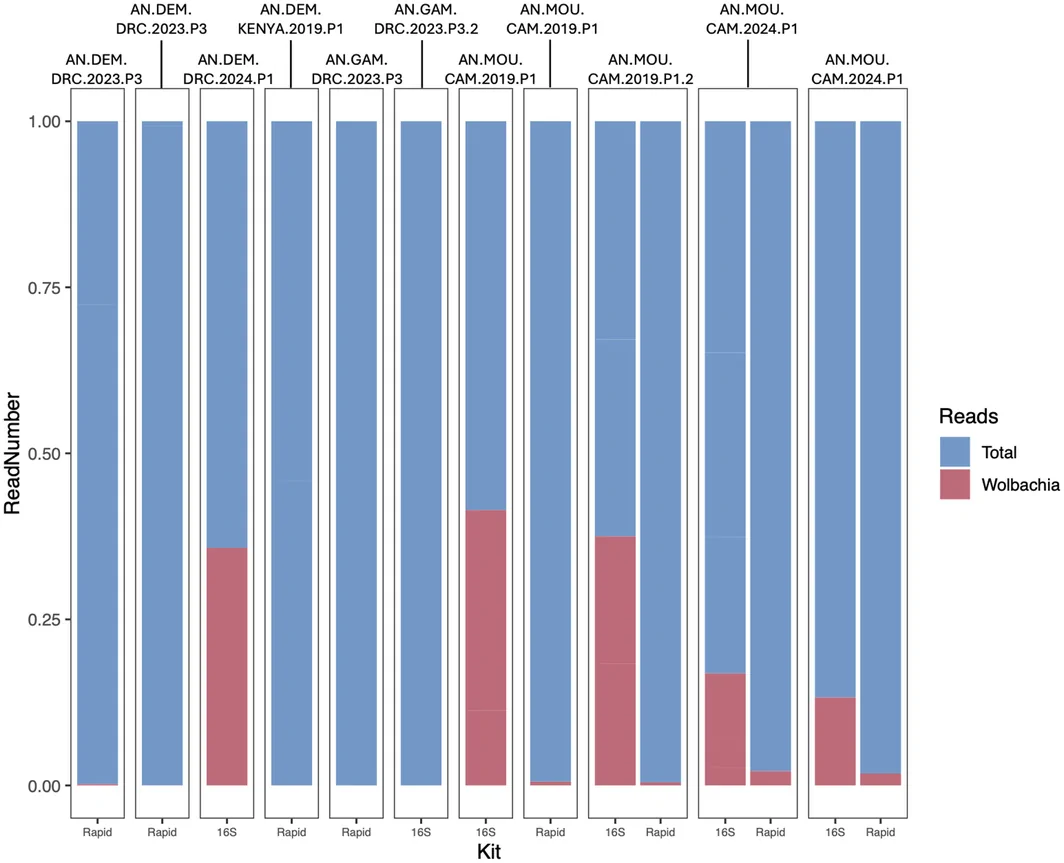
Proportion of total reads assigned to *Wolbachia* from ONT Flonge flow cell sequencing. Libraries prepared and sequenced using either the Rapid PCR Barcoding Kit 24 V14 (Rapid) or 16S Barcoding Kit 24 V14 (16S).

## 4 Discussion

Overall, ONT sequencing using libraries prepared with the field sequencing kit and run on MinION flow cells resulted in a higher proportion of reads called and a greater amount of passed called bases than the Flongle flow cell runs using either the Rapid PCR Barcoding Kit or 16S Barcoding Kit library preparation method. However, ONT sequencing on the Flongle flow cells was able to generate similar numbers of estimated bases, reads generated and total data produced. Our results suggest that if the main data output desired is detection of *Wolbachia* in *Anopheles* using Flongle flow cells then the 16S Barcoding Kit library preparation method is optimal given this averaged 2038 *Wolbachia* reads across *Anopheles* species (*An. demeilloni* and *An. moucheti*) that have previously been shown to have *Wolbachia* strains in genuine symbiosis (Walker et al. 2021). This was expected as the Rapid PCR Barcoding Kit libraries result in both the mosquito and microbiome rather than the 16S Barcoding Kit which targets just the bacterial microbiome.

The 16S Barcoding Kit library preparation method was also able to provide consistent microbiome profiles in which *Wolbachia* was the dominant species which is consistent with previous profiles using *16S rRNA* Illumina amplicon sequencing (Walker et al. 2021). The absence of *Wolbachia* reads in *An. gambiae* from flongle flow cell ONT sequencing is also consistent with previous Illumina sequencing and PCR based studies not providing conclusive evidence for the presence of a genuine natural *Wolbachia* strain in this species (Walker et al. 2021; Sawadogo et al. 2022). However, *Asaia* was detected in *An. gambiae* (sample AN.GAM.DRC.2023.P3) at a high relative abundance, accounting for over 50% of the reads, providing further evidence for a potential competitive mutual exclusion between these two bacteria. *Wolbachia* strains in *Anopheles* have been shown to be inversely correlated to *Asaia* which is stably associated with several *Anopheles* species (Wickham 2016; Favia et al. 2007; Rossi et al. 2015; Jeffries et al. 2018, 2021). There are limited studies reporting co‐infections in wild *Anopheles* populations (Jeffries et al. 2018) but there were small numbers of individual co‐infections from *An. melas* and *An. gambiae* s.s.–*melas* hybrids collected in Guinea (Jeffries et al. 2021). Due to the limited number of samples capable of being run on a single flongle flow cell, we were unable to assess run‐to‐run variation of *Wolbachia* counts due to the high levels of inter‐individual differences often seen between individual mosquitoes.

ONT sequencing has been used previously in field settings due to its portability, price and ease of use for local/in‐field sequencing. For example, in 2015 ONT was used to sequence samples that contained Ebola virus during the largest outbreak on record (Quick et al. 2016). The use of ONT for vector‐borne disease surveillance is increasing with ONT sequencing of *Pl. falciparum* malaria parasites from dried blood spots has been developed that can detect key drug‐resistance associated genes, diagnostic test antigens and polymorphic markers (de Cesare et al. 2024; Adegbola et al. 2024; Hosch et al. 2024; De Meulenaere et al. 2024). Mosquito genome sequencing is also possible as ONT contributed to the development of an improved chromosome‐scale genome assembly for *Culex quinquefasciatus* (the principal vector of West Nile virus) (Ryazansky et al. 2024) and produced de novo chromosome‐level genome assemblies for *An. coluzzii* and *An. arabiensis (*Zamyatin et al. 2021 *)*. It could furthermore be established using dried bloodspots screening for antimalarial resistance markers in resource‐limited laboratory settings (Girgis et al. 2023). Mosquito bloodmeal analysis has been undertaken using ONT (Kothera et al. 2024) and ONT sequencing has also been tested for arbovirus surveillance (Mirza et al. 2024) including to identify yellow fever virus (YFV) in mosquitoes collected from Brazil during an outbreak in 2021 (de Oliveira et al. 2023). Ross River virus screening was undertaken on *Aedes* mosquito RNA in 2017 but required the use of MinION flow cells and was less accurate compared to illumina amplicon sequencing due to a high error rate (de Vries et al. 2023). Large scale ONT sequencing (using flongle flow cells) on *Anopheles* mosquitoes analysed over 6000 samples collected between 2003 and 2019 for the presence of Bagaza virus (Sekee et al. 2024).

There are some methodological limitations to the experimental design employed in this study that should be considered when interpreting results. Firstly, due to issues with amplification, the number of PCR cycles was increased from the recommended 14 to 25 cycles for the Rapid Barcoding library preparation. This was due to PCR success being determined by the presence and intensity of the resulting amplicon using agarose gel electrophoresis. This may increase the potential of non‐specific amplification and chimera formation. The bioinformatics pipeline employed did not have a dedicated chimera removal step and so this cannot be definitively excluded. However, the inclusion of an *Anopheles gambiae* sample that is known to be negative for *Wolbachia* infection was processed using the same methodology and produced no *Wolbachia*‐assigned reads. This is reassuring that the study design did not generate false‐positive *Wolbachia* reads. Future work should include chimera removal within the bioinformatics pipeline and include technical replication between sequencing runs to rule out this potential limitation. Variation in *Wolbachia*‐assigned read counts was seen between sequencing runs and individual samples; this may be due to a number of factors including concentration and quality of starting genetic material, number of pores available on each flow cell and genuine differences in *Wolbachia* infection density between samples which has been seen in previous studies (Walker et al. 2021). As this study was not designed to explore the impact of biological and technical variation, the methodology presented here should be primarily used for qualitative *Wolbachia* detection in field samples, rather than accurate quantification of *Wolbachia* infection density.

For detecting *Wolbachia* strains in *Anopheles* mosquitoes, the 16S Barcoding Kit does have limitations for use in field settings such as the need for laboratory equipment for DNA extraction and library preparation. *Wolbachia* has recently been detected using ONT in fleas and ticks ( *Rhipicephalus sanguineus* and *Ctenocephalides felis felis*) (Paez‐Triana et al. 2025) using the Ligation sequencing gDNA kit (SQK‐LSK109). In addition, ONT sequencing from libraries prepared using the Ligation sequencing gDNA kit has been used to identify a *Wolbachia* bacteriophage (phage WO) of the southern ground cricket ( *Allonemobius socius* ) (Kupritz et al. 2021) but unfortunately this kit has been discontinued limiting comparison to our study. Despite ONT having the advantage of requiring less infrastructure than other methods of sequencing, to generate libraries using the 16S barcoding kit the minimum equipment needed is a thermocycler, hula mixer, magnetic racks and vortex machines. The flongle flow cells run on the MinION Mk1B device also require significant computing power, with a minimum storage requirement of 1 TB, which may not be achievable in field settings. The barcoding kits used allowed for the testing of a theoretical maximum of 24 samples but due to limitations in read numbers we found only four *Anopheles* mosquito samples could be used per flongle flow cell run limiting sample numbers.

## 5 Conclusions

Malaria disproportionately affects the WHO African Region that is also facing great economic challenges. A more economically viable ‘in‐field’ microbiome sequencing method may be crucial to help develop novel vector control strategies such as those using *Wolbachia* bacteria. Though not employed in the current study, ONT also has the advantage of variable read lengths and ultra‐long reads (> 1 Mb) permitting whole genome sequencing. This could provide a rapid method of sequencing *Wolbachia* strain genomes that are present in wild *Anopheles* populations as to date only two strains have been sequenced (Quek et al. 2022). ONT sequencing quality could be further improved by extraction of *Anopheles* DNA using kits that target high molecular weight DNA.

## Author Contributions

**Laura Chatterley:** investigation, formal analysis, methodology, writing – review and editing, visualisation, data curation, project administration, writing – original draft, resources. **Nicola Hull:** investigation, writing – original draft, visualisation, data curation, formal analysis, resources. **Isabel Hughes:** resources, investigation, writing – review and editing. **Seynabou Sougoufara:** resources, investigation, writing – review and editing. **Owain Meek:** resources, investigation, writing – review and editing. **Murat Ceyran:** resources, investigation, writing – review and editing. **Vishaal Dhokiya:** resources, investigation, writing – review and editing. **Janvier Bandibabone:** resources, investigation, writing – review and editing. **Cyprian Adala:** resources, investigation, writing – review and editing. **Elin Cunningham:** resources, investigation, writing – review and editing. **Shehu Shagari Awandu:** resources, investigation, writing – review and editing. **Helene Kaminski‐Nenkam:** resources, investigation, writing – review and editing. **Antonio Nkondjio:** resources, investigation, writing – review and editing. **Grant L. Hughes:** supervision, funding acquisition, resources, investigation, writing – review and editing. **Eva Heinz:** writing – review and editing, methodology, supervision, funding acquisition, visualisation, validation, investigation. **Thomas Walker:** conceptualisation, writing – original draft, funding acquisition, supervision, resources, writing – review and editing, methodology, project administration, visualisation, investigation, data curation, validation.

## Funding

This work was supported by the Bill and Melinda Gates Foundation (INV‐048598), the Open Philanthropy via Anti‐Vec, the Biotechnology and Biological Sciences Research Council (UKRI543:2023BBSRC‐NSF/BIO, BB/V011278/1, BB/X018024/1, BB/W018446/1, BB/V011278/2), the National Health and Medical Research Council (2038930), the Sir Henry Dale Wellcome Trust Royal Society Fellowship (101285/Z/13/Z), the UK Research and Innovation (20197), the National Institute for Health and Care Research (NIHR2000907) and the African Research Excellence Fund (AREF‐312‐AWAN‐F‐C0906, AREF‐312‐AWAN‐S‐C1027).

## Ethics Statement

Ethical approval for undertaking Human landing catches (HLCs) in Cameroon was obtained by LSTM ethics committee (reference no. 16684) in addition to local ethical approval (clearance no. 2016/01/685/CE/CNERSH/SP) delivered by the Cameroon National Ethics (CNE) Committee for Research on Human Health for the 2019 collections. For the 2022 and 2024 collections, ethical approval (clearance no. 02060/CRERSHC/2022) was delivered by the Cameroon National Ethics (CNE) Committee for Research on Human Health. Informed consent was gained from all collectors prior to the commencement of sampling and all collectors were provided with malarial chemoprophylaxis. Ethical clearance was also obtained for HLCs in Kenya (KEMRI/SERU/CGHR/317/3919) via The KEMRI Scientific and Ethics Review Unit (SERU). No ethical approval was required for work carried out in the DRC as no HLCs were used.

## Conflicts of Interest

The authors declare no conflicts of interest.

## Data Availability

The data that support the findings of this study are openly available in NCBI Sequence Read Archive (SRA) at https://www.ncbi.nlm.nih.gov/bioproject/PRJNA1411912, reference number PRJNA1411912.
